# ANCA-associated vasculitis—treatment standard

**DOI:** 10.1093/ndt/gfad237

**Published:** 2023-11-08

**Authors:** Aglaia Chalkia, David Jayne

**Affiliations:** Department of Medicine, University of Cambridge, Cambridge, UK; Nephrology Department, Hippokration General Hospital, Athens, Greece; Department of Medicine, University of Cambridge, Cambridge, UK

**Keywords:** ANCA-associated vasculitis, diagnosis, pathophysiology, prognosis, treatment

## Abstract

Anti-neutrophil cytoplasmic antibody (ANCA)-associated vasculitides (AAV) are characterized by small-vessel necrotizing inflammation, and prior to the advent of immunosuppressive therapy frequently had a fatal outcome. Treatment has transformed AAV into a relapsing/remitting disease with increased drug-related toxicities and organ damage. The use of glucocorticoids, cyclophosphamide and immunosuppressives (including azathioprine, mycophenolate and methotrexate) was optimized through a sequence of clinical trials establishing a standard of care against which subsequent targeted therapies could be developed. Improved understanding of pathophysiology has supported the development of B-cell depletion and complement inhibition in granulomatosis with polyangiitis and microscopic polyangiitis, and interleukin 5 inhibition for eosinophilic granulomatosis with polyangiitis, leading to the approval of newer agents for these conditions. There has been an increased attention on minimizing the adverse effects of treatment and on understanding the epidemiology of comorbidities in AAV. This review will focus on recent evidence from clinical trials, especially with respect to glucocorticoids, avacopan, plasma exchange, rituximab and mepolizumab, and their interpretation in the 2022 management recommendations by the European League of Associations of Rheumatology.

**In a nutshell**

1. Induction treatment of life/organ-threatening anti-neutrophil cytoplasmic antibody–associated vasculitis is a combination of glucocorticoids and rituximab or cyclophosphamide, with rituximab the preferred choice in relapsing granulomatosis with polyangiitis/microscopic polyangiitis.
2. A rapidly reducing glucocorticoid regimen is now preferred—the Plasma Exchange and Glucocorticoids in Severe ANCA-Associated Vasculitis (PEXIVAS) Trial schedule—which reduces risk of serious infection without loss of efficacy. Although plasma exchange did not improve the combined endpoint of death and/or end-stage kidney disease (ESKD), a meta-analysis concluded that plasma exchange results in reduced risk of ESKD at 12 months and should be considered in patients presenting with a serum creatinine >300 μmol/L.
3. For maintenance of remission treatment, fixed-interval repeat-dose rituximab for 24–48 months is more effective than azathioprine or methotrexate and permits glucocorticoid discontinuation within 6 months of start of therapy. Relapse risk increases after rituximab withdrawal. An understanding of the risks and consequences of relapse and the risks of secondary immunodeficiency with rituximab informs the physician's decision on treatment duration.
4. New insights into the role of the complement alternative pathway in pathogenesis led to the development of the oral anti-C5a receptor, avacopan, which was superior over 1 year to a standard glucocorticoid tapering regimen, when given in combination with rituximab or cyclophosphamide. Notably, avacopan led to more rapid reduction in proteinuria and improved kidney recovery.
5. The anti-interleukin 5 agent, mepolizumab, has permitted glucocorticoid reduction, improved remission and reduced relapse rates in eosinophilic granulomatosis with polyangiitis.

## INTRODUCTION

Historically, untreated anti-neutrophil cytoplasmic antibody (ANCA)-associated vasculitis (AAV) had a 1-year mortality of 80% [1]. The introduction of glucocorticoids prolonged survival but required the combination with cyclophosphamide to achieve stable remission [2]. The toxicity of prolonged cyclophosphamide encouraged the use of safer oral immunosuppressives and limiting cyclophosphamide use to an induction period of 3–6 months. While cyclophosphamide was established as the routine immunosuppressive for induction, alternative immunosuppressive, methotrexate and mycophenolate, were tested but with limited success. Rituximab, introduced in the early 2000s, was the first effective alternative to cyclophosphamide. It is favoured for relapsing disease and increasingly selected as a first-line induction agent, with its use now extended to the longer term prevention of relapse.

AAV is heterogeneous at presentation with respect to organ involvement and severity and this has led to attempts to subgroup patients based on clinical presentation, to tailor choice of treatment. No system of subgrouping has become widely accepted and this remains a confusing area with terminology, such as severe/non-severe, major/minor and organ threatening/non-organ threatening, used without robust definitions. The goals of treatment is to achieve remission, and delayed remission or failure to achieve remission is reflected in higher mortality and damage, including end-stage kidney disease (ESKD) risks [3]. Once remission is achieved the next goal is to prevent relapse, and over 50% of AAV patients will relapse during the course of their disease despite ongoing maintenance therapy.

Most patients survive their disease presentation with some irreversible organ damage exacerbated by further episodes of vasculitic relapse. The accrual of damage, prevention of relapse and development of comorbidities, especially infection, cardiovascular disease and cancer, dominate long-term patient management with important implications for treatment selection and monitoring [4].

## TREATMENT STANDARDS

**Figure 1:**
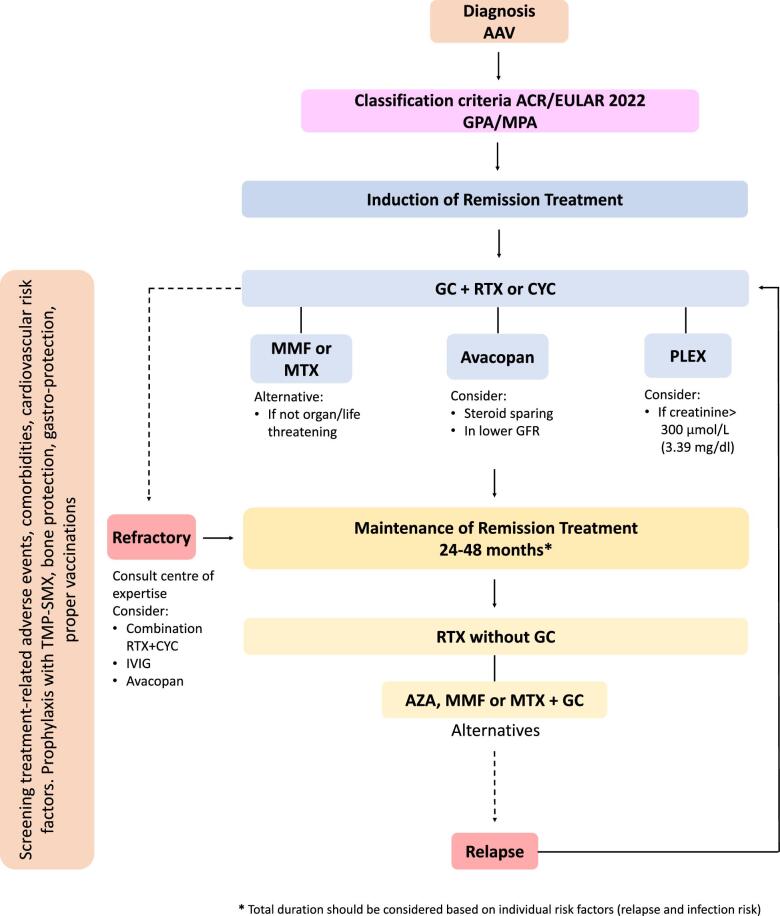
Proposed therapeutic algorithm for the management of GPA and MPA. GC, glucocorticoid; RTX, rituximab; CYC, cyclophosphamide; MMF, mycophenolate mofetil; MTX, methotrexate; IVIG, intravenous immunoglobulin; AZA, azathioprine; TMP-SMX, trimethoprim/sulfamethoxazole.

**Figure 2:**
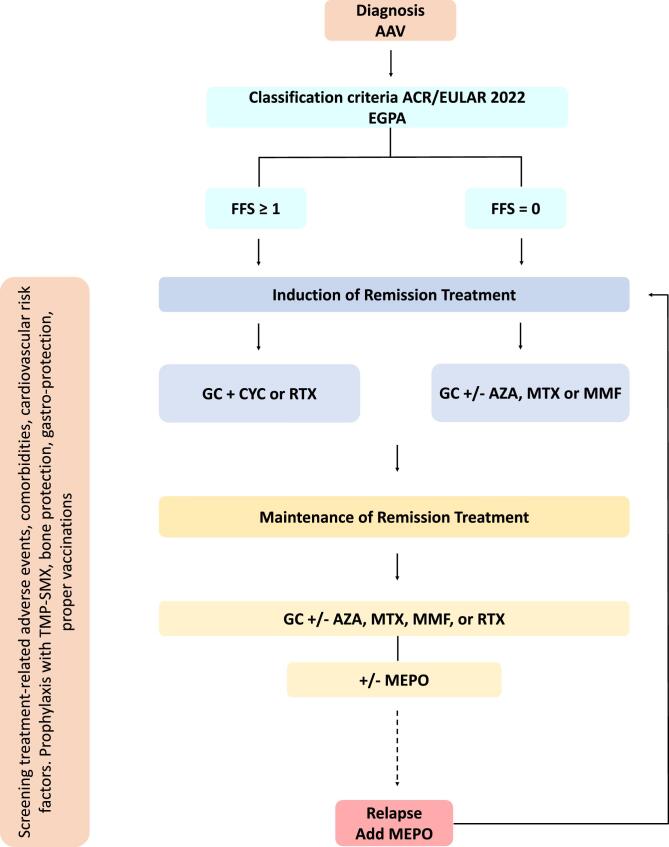
Proposed therapeutic algorithm for the management of EGPA. GC, glucocorticoid; RTX, rituximab; CYC, cyclophosphamide; AZA, azathioprine; MTX, methotrexate; MMF, mycophenolate mofetil; MEPO, mepolizumab; TMP-SMX, trimethoprim/sulfamethoxazole.

AAV are divided into three clinical phenotypes of granulomatosis with polyangiitis (GPA) and microscopic polyangiitis (MPA), which are typically combined for clinical studies given their similar initial responses to standard therapy, and eosinophilic granulomatosis with polyangiitis (EGPA) [5] (Figs 1 and 2). Although this review focuses on the 2022 management recommendations by the European League of Associations of Rheumatology (EULAR), the American College of Rheumatology (ACR) jointly with the Vasculitis Foundation (VF) and Kidney Disease: Improving Global Outcomes (KDIGO) have also updated their guidelines [5–7].

## TREATMENT IN GPA/MPA

Induction therapy for GPA/MPA is glucocorticoids and either rituximab or cyclophosphamide [8–10]. Variance to this approach can be considered in particular patient scenarios. Rituximab is preferred for relapsing disease based on data from the Rituximab versus cyclophosphamide for ANCA-Associated Vasculitis (RAVE) and rituximab versus azathioprine for maintenance of remission for patients with ANCA-associated vasculitis and relapsing disease (RITAZAREM) trials [9, 11]. The combination of rituximab with low-dose cyclophosphamide was as effective as full-dose cyclophosphamide in the Rituximab versus Cyclophosphamide in ANCA-Associated Renal Vasculitis (RITUXVAS) Trial [8] and this approach has been effective and steroid-sparing in observational studies especially for patients with rapidly progressive glomerulonephritis, but has not been formally compared with rituximab without cyclophosphamide and carries a higher risk of immune suppression [12, 13]. In practice, if one of rituximab or cyclophosphamide is selected at the beginning of induction treatment and there is not an optimal response then the other can be added. Intravenous cyclophosphamide is preferred to oral cyclophosphamide due to reduced total drug exposure and much reduced risks of bladder toxicity (Table 1).

**Table 1:** Drug therapies commonly used and/or approved for the treatment of AAV.

Drug	Dose	Marker of response	Precautions	Toxicity
Glucocorticoids	Induction [16]	CRP/ESR	Cautious use in diabetes mellitus or obesity	Cardiovascular
	• IV 1–3 g (on Days 1–3) • Oral 1 mg/kg/day (prednisolone equivalent) • Tapering according to ‘PEXIVAS reduced schema’; see Table 2	BVAS	Prophylaxis TMP-SMX (at a dose of ≥30 mg/day for ≥4 weeks)	Endocrine/metabolic Infections Gastrointestinal Dermatological Musculoskeletal Psychological Ophthalmological
			Prophylaxis with calcium/vitamin D	
			Prophylaxis with PPI	
			Safety in pregnancy	
Cyclophosphamide	Induction [8, 9, 57]	CRP/ESR	Dose reduction	Infertility
	• IV 15 mg/kg at Weeks 0, 2, 4, 7, 10, 13 (16, 19, 21, 24 if required) • Oral 2 mg/kg/day for 3 months (maximum 6 months)	BVAS	-Age >60 years - GFR <30 mL/min/1.73 m^2^ Check FBC Removal by haemodialysis Prophylaxis TMP-SMX Should be avoided for the first 10 weeks of gestation	Malignancy Bone marrow suppression Infections Urinary bladder toxicity
Rituximab	Induction [8, 9]	CRP/ESR	Check IgG levels	HBV reactivation
	• IV 1000 mg at Weeks 0 and 2	BVAS	Check HBV (HBsAg and anti-HBcAb)	Hypogammaglobulinemia
	• IV 375 mg/m^2^ once weekly for 4 doses	CD19 B cells	Check TB	Lower response to vaccinations
	Maintenance [11, 23]	ANCA levels	Removal by PLEX	Infections
	• IV 500 mg 0 and 2 weeks and then 6-monthly for 24–48 months		Prophylaxis TMP-SMX Should be avoided in third trimester of gestation	PML
	• IV 1000 mg 4-monthly could be considered			
Cyclophosphamide–rituximab combination	Induction [8, 12]	CRP/ESR	See ‘rituximab’, ‘cyclophosphamide’	See ‘rituximab’, ‘cyclophosphamide’
	MPA/GPA	BVAS		Higher infections
	• Rituximab IV 1000 mg at Week 0 and 2 or IV 375 mg/m^2^ once weekly for 4 doses			
	• Cyclophosphamide IV 15 mg/kg at weeks 0 and 2			
	Or alternative			
	• Rituximab IV 1000 mg at Week 0 and 2			
	• Cyclophosphamide IV 500 mg/2weeks ×6			
Mycophenolate mofetil	Induction/maintenance [15]	CRP/ESR	Check FBC	Bone marrow suppression
	• Oral 2000 mg/day (divided doses, may be increased to 3000 mg/day)	BVAS	Check liver function tests	Teratogenicity
			Contraindication in pregnancy	Hepatotoxicity
				Infections
				Gastrointestinal toxicity
Methotrexate	Induction/maintenance [14] • Oral/sc 15 mg/week initial (maximum 20–25 mg/week)	CRP/ESR BVAS	Check kidney function (dose reduction dose at GFR 30–60 mL/min and discontinuation at GFR <30 mL/min)	Bone marrow suppression Kidney toxicity Liver toxicity Gastrointestinal toxicity Photosensitivity Pulmonary toxicity
			Check liver function	
			Check FBC	
			Prophylaxis with folic acid (5 mg/week)	
			Contraindication in pregnancy	
Avacopan	Induction [10]	CRP/ESR	Check liver function tests	HBV reactivation
	MPA/GPA	BVAS	Check WBC	Hepatotoxicity
	• Oral 30 mg twice per day	SF-36	Check HBV (HBsAg and anti-HBcAb)	Nausea
		Proteinuria	Check TB	Headache
			Unknown safety in pregnancy	Leukopenia
				Unknown safety beyond 1 year
Mepolizumab	Induction/maintenance	Eosinophils	Unknown safety in pregnancy	Hypersensitivity reactions
	EGPA	CRP/ESR		
	• sc 300 mg every 4 weeks [29] or alternative	BVAS		
	• sc 100 mg every 4 weeks (approved dose for treating severe eosinophilic asthma) [30]			
Azathioprine	Maintenance [11, 23, 24]	CRP/ESR	Check TPMT	Bone marrow suppression
	• Target oral 2 mg/kg/day	BVAS	Check FBC	Hepatotoxicity
			Check liver function tests	Gastrointestinal toxicity
			Reduce sun exposure	Skin malignancy
			Safety in pregnancy	
Intravenous Immunoglobulin	Refractory disease [58]	CRP/ESR	Check serum creatinine	Headache
	• IV 0.4 g/kg/day for 5 days (total dose 2 g/kg)	BVAS	Check blood pressure	Aseptic meningitis
			Use with caution in elderly patients	Reversible rises in serum creatinine
				Thromboembolic events

IV, intravenous; CRP, C-reactive protein; ESR, erythrocyte sedimentation rate; BVAS; Birmingham Vasculitis Activity Score; FBC, full blood count; TMP-SMX, trimethoprim/sulfamethoxazole; PPI, proton pump inhibitors; IgG, immunoglobulin G; HBV, hepatitis B virus; HBsAg; HBV surface antigen; anti-HBcAb, HBV core antibody; TB, tuberculosis; PML, progressive multifocal leukoencephalopathy; SF-36, 36-Item Short Form Survey; WBC, white blood cell, sc, subcutaneous; TPMT, thiopurine methyltransferase testing.

Methotrexate has been considered an option for patients without organ-threatening GPA, but relapse rates were high and most patients subsequently required cyclophosphamide [14]. Mycophenolate mofetil was also associated with a higher relapse rate in GPA but may have a role for carefully selected MPA patients without evidence of rapid loss of kidney function [15]. However, both developments used higher doses of glucocorticoid than currently recommended which will have contributed to the apparent benefit (Tables 1 and 2) [16].

**Table 2:** Glucocorticoid dosing (mg/day, prednisolone equivalent) with rituximab- or cyclophosphamide-based regimens for remission induction according to the PEXIVAS study [16].

Week	<50 kg	50–75 kg	>75 kg
1	50	60	75
2	25	30	40
3–4	20	25	30
5–6	15	20	25
7–8	12.5	15	20
9–10	10	12.5	15
11–12	7.5	10	12.5
13–14	6	7.5	10
15–18	5	5	7.5
19–52	5	5	5
>52	Individual taper	Individual taper	Individual taper

The results of the PEXIVAS trial [16] conducted in 704 AAV patients with glomerular filtrate rate (GFR) <50 mL/min/1.73 m^2^ or lung haemorrhage did not demonstrate a significant delay in the time to the composite endpoint of death or ESKD, but subsequent meta-analysis [17] confirmed that plasma exchange (PLEX) reduced the risk of ESKD at 12 months in subgroups, based on serum creatinine, with higher risk of ESKD. Thus, PLEX (7 sessions within 14 days) can be considered for patients presenting with a serum creatinine >300 µmol/L (3.39 mg/dL) [18]. AAV patients with lung haemorrhage and hypoxia at presentation in the PEXIVAS trial showed a trend to reduced mortality with plasma exchange [19]. An increased risk of infection with plasma exchange was observed in the meta-analysis and this risk needs to be balanced against potential benefits.

Dosing and duration of glucocorticoids had been partially standardized in the design of early clinical trials in AAV, but direct comparison of different glucocorticoid regimens has now been reported from the PEXIVAS and Effect of Reduced-Dose vs High-Dose Glucocorticoids Added to Rituximab on Remission Induction in ANCA-Associated Vasculitis (LOVAS) trials [16, 20]. The PEXIVAS trial demonstrated similar efficacy for a reduced-dose oral glucocorticoid regimen (Table 2) compared with a standard glucocorticoid regimen (cumulative exposure difference between groups of 40%) for the primary endpoint (ESKD or death) but fewer serious infections occurred with the reduced dose regimen. The majority of PEXIVAS patients received cyclophosphamide induction and it is not clear whether the reduced-dose glucocorticoid regimen should be recommended with rituximab as dosing was lower than that used in the RAVE trial. In the Avacopan for the Treatment of ANCA-Associated Vasculitis (ADVOCATE) Trial, when compared with a glucocorticoid regimen similar to the reduced dose PEXIVAS regimen but with withdrawal at 21 weeks, avacopan led to more patients having sustained remission at 12 months and showed superiority for improvement in quality of life, recovery of GFR and fewer glucocorticoid-related complications [10]. It is therefore an alternative to glucocorticoids, attractive for those patients at high risk of glucocorticoid toxicity. Notably, in ADVOCATE among the patients with renal involvement (81%), the avacopan-treated group experienced a more rapid improvement in the urine albumin–creatinine ratio by the first month [absolute difference –40% (–53% to –22%)] and higher GFR recovery until 52 weeks (mean difference 3.2 mL/min/1.73 m^2^). The differences in GFR recovery were more pronounced in patients with lower GFR (mean difference 5.5 mL/min in GFR <30 mL/min, 8.4 mL/min in GFR <20 mL/min), which was sustained during the 8-week follow-up period after discontinuation [21]. However, there is no clinical trial data to support the use of avacopan for patients with GFR <15 mL/min/1.73 m^2^ or for longer term therapy beyond 1 year. Intravenous methylprednisolone at cumulative doses between 1000 mg and 3000 mg is widely used for patients presenting with organ-threatening disease but has not been tested in a randomized clinical trial (Table 1).

The failure of a patient to respond to induction therapy or to achieve a complete remission defines refractory disease [22]. Alternative diagnoses, or the presence of a vasculitis secondary to another disease process (malignancy, infection, drugs, e.g. cocaine) need to be considered. Instability in control of disease activity early in the treatment course is not infrequent and is managed by an increase in oral, or use of intravenous, glucocorticoids. Other options are to combine cyclophosphamide and rituximab, to introduce avacopan or consider PLEX. High-dose intravenous immunoglobulins has also been used especially in the context of concurrent infection.

The first choice for maintenance of remission treatment after induction with rituximab or cyclophosphamide has changed to rituximab from azathioprine or methotrexate following the Rituximab versus Azathioprine for Maintenance in ANCA-Associated Vasculitis (MAINRITSAN) and RITAZAREM results [11, 23]. These trials studied different patient populations and different rituximab doses, but a dose of 500 mg every 6 months for 2–4 years is now recommended, although some patients may require a higher/more frequent dose (Table 1). Extending the remission treatment period from 2 to 4 years has been supported by data with azathioprine and prednisolone from the Randomised controlled trial of prolonged treatment in the remission phase of ANCA-associated vasculitis (REMAIN) Trial [24] and from prolonged rituximab use in the Long-Term Rituximab Use to Maintain Remission of Antineutrophil Cytoplasmic Antibody-Associated Vasculitis (MAINRITSAN III) Trial [25]. Frequent complications of rituximab are infections and hypogammaglobulinemia, which can be severe, requiring prolonged infection prophylaxis and immunoglobulin replacement therapy. Immunoglobulin G levels should be monitored every 6 months and a falling level, such as below 5 g/L, requires reassessment of risk/benefit for continuing rituximab treatment. Patient factors including the perceived relapse risk, the possible consequences of relapse and the risks of ongoing treatment toxicity need to be reviewed during the course of maintenance therapy. Azathioprine, methotrexate and mycophenolate mofetil are alternatives to rituximab and are often combined with low-dose glucocorticoids (Table 1). Glucocorticoid withdrawal increases relapse risk in this setting.

## TREATMENT IN EGPA

The Five-Factor Score (FFS; impaired kidney function, proteinuria, cardiomyopathy, gastrointestinal tract and central nervous system involvement, each 1 point if present) defined organ involvement that predicted mortality in EGPA, and a FFS ≥1 is used to define poor prognosis patients. For these patients the recommendation is high dose of glucocorticoids and cyclophosphamide as initial treatment. A recent prospective study of 70 patients with EGPA and cardiac involvement treated by glucocorticoid and cyclophosphamide, also reported favourable long-term prognosis [26]. The Rituximab versus Conventional therapeutic strategy for remission induction in eosinophilic granulomatosis with polyangiitis (REOVAS) Trial randomized 105 EGPA patients (40% FFS ≥1) to rituximab or cyclophosphamide and reported that rituximab was not inferior for remission induction at 6 months (abstract) [27] with a high-dose glucocorticoid tapering regimen. In one study, Adding Azathioprine to Remission-Induction Glucocorticoids for Eosinophilic Granulomatosis With Polyangiitis (Churg-Strauss), Microscopic Polyangiitis, or Polyarteritis Nodosa Without Poor Prognosis Factors (CHUSPAN) Trial for EGPA patients with a FFS of 0, the addition of azathioprine to glucocorticoids did not reduce relapse risk [28]. However, relapses in EGPA are frequent as glucocorticoids are reduced and most patients accrue a high glucocorticoid exposure. As a result, other immunosuppressives are still often used, to try to limit glucocorticoid exposure. The anti-interleukin 5 (IL-5) monoclonal antibody mepolizumab dosed at 300 mg every 4 weeks was effective in prevalent EGPA patients, where it reduced steroid exposure, improved remission rates and reduced relapse risk in the Mepolizumab or Placebo for Eosinophilic Granulomatosis with Polyangiitis (MIRRA) Trial [29]. The mepolizumab dose approved for treating severe eosinophilic asthma (100 mg every 4 weeks) may also be considered for EGPA [30]. Randomized trials evaluating optimal mepolizumab dose in EGPA have not been performed. In the EGPA MIRRA trial, much of the improvement reflected control of asthma and naso-sinus disease, and there are insufficient data to recommend use of mepolizumab in severe vasculitic presentations with organ failure. The benefit of anti-IL-5 is seen in both ANCA-negative and -positive EGPA subgroups, and is maintained beyond 1 year of treatment. Benefit has also been reported with rituximab in observational studies of prevalent EGPA patients with relapsing/refractory disease [31]; however, the inference across studies is that rituximab is not as beneficial in EGPA compared with GPA/MPA.

## MANAGING INFECTION, TREATMENT-RELATED COMPLICATIONS AND COMORBIDITIES

Infective prophylaxis for pneumocystis with trimethoprim/sulfamethoxazole is recommended when cyclophosphamide or rituximab and/or high doses of glucocorticoids are used, and appears to have a more general effect on reducing bacterial infection frequency [32]. Response to immunization is markedly impaired following rituximab and there is rarely the opportunity to immunize prior to induction therapy [33]. However, vaccination timing can be optimized during rituximab maintenance therapy, ideally administering vaccines at least 4 weeks before the next rituximab dose. Fundamental principles in the management of AAV patients also involve the periodically screening for and management of cardiovascular factors, such as diabetes, lipids, blood pressure and other treatment-related complications, including osteoporosis, guided by general EULAR recommendations [34, 35]. Addressing the distinctive characteristics of AAV patients, including vulnerable demographics like the elderly, who are more susceptible to higher infection rates and treatment-associated toxicities, and younger patients of reproductive age, the notion of tailoring treatments considering the treatment toxicity profile (Table 1) could be incorporated.

Box. **Strategies for personalizing treatment in AAV.**

**Personalizing treatment based on patients characteristics**

- Patients with severe kidney disease
- Consider addition of plasma exchange (if serum creatinine >300 μmol/L)
- Consider addition of avacopan for lower GFR

- Elderly/frail patients (vulnerable to myelotoxicity and infections)
- Consider rituximab over cyclophosphamide
- Consider steroid-sparing strategy with avacopan

- Pre-menopausal women and men concerning their fertility
- Consider rituximab over cyclophosphamide

- Patients with relapsing disease
- Consider rituximab (MPA/GPA)
- Consider mepolizumab (EGPA)

**Personalized treatment based on choice of treatment**

- Combination cyclophosphamide and rituximab
- Severe kidney disease
- Slow disease response
- Refractory disease
- High risk of glucocorticoid toxicity (steroid-sparing strategy)
- High risk of cyclophosphamide toxicity (cyclophos-phamide-sparing strategy)

- Addition of avacopan
- Glucocorticoids contraindicated/high risk of toxicity
- Lower GFR
- Refractory disease
- After starting avacopan, consider rapid glucocorticoid taper and withdrawal by end of Week 4
- Duration 12 months

## NEW DEVELOPMENTS

### Pathophysiology

Genetic factors contribute to the pathogenesis of AAV. Genetic variants, both within the major histocompatibility complex (MHC) and non-MHC genes, associate with AAV susceptibility and disease characteristics. MHC variants display differential associations between proteinase 3 (PR3)-ANCA and myeloperoxidase (MPO)-ANCA AAV subtypes [36]. Both serotypes are associated with variants of SERPINA1 (encodes for alpha 1 anti-trypsin), a key inhibitor of the serine protease PR3, PTPN22 (protein tyrosine phosphatase non-receptor type 22) and CTLA4 (cytotoxic T lymphocyte antigen 4) which play roles in T cell activation [37]. A genome-wide association study has indicated associations between HLA-DQ and MPO-ANCA-positive EGPA, while ANCA-negative EGPA is related to variants associated with mucosal responses and eosinophil biology [38].

The success of B-cell depletion therapy has been attributed to the recognition of the central role of B cells in the pathogenesis of AAV, given their key roles of ANCA-producing plasma cells and their function as antigen-presenting cells that support T-cell activation and possibly other cell types, especially in granulomatous lesions. Specific B-cell cytokines, especially B cell–activating factor/lymphocyte stimulator (BAFF/BLyS), have emerged as a crucial factor in the development and function of B cells. BAFF levels correlate with AAV disease activity and BAFF/BLyS receptor antagonists are potential treatment targets [39].

**Figure 3:**
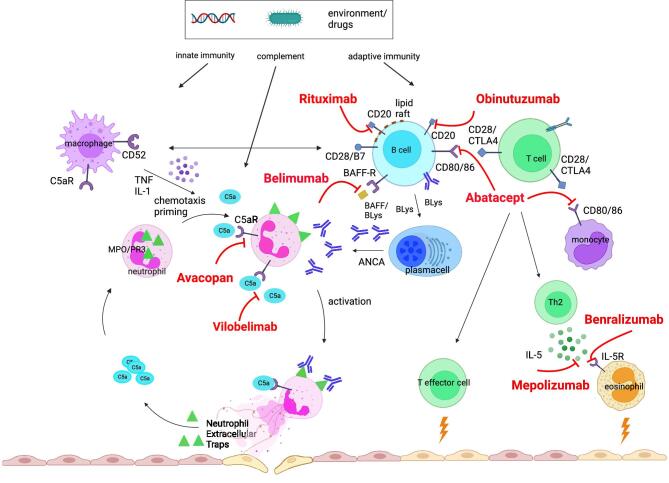
Pathogenesis of AAV and potential targeted therapies. TNF, tumor necrosis factor alpha; IL-1, interleukin-1; IL-5R, interleukin 5 receptor; Th2, T helper 2 cells; avacopan, target C5aR; vilobelimab, target C5a; belimumab, target BAFF/Blys; rituximab, target CD20; obinutuzumab, target CD20; abatacept, ligand-binding domain of CTLA4; mepolizumab, target IL-5; benralizumab; target IL-5R.

The complement alternative pathway in AAV has been shown in experimental models to be a critical pathway for ANCA vasculitis and *in vivo* complement activation through increased serum levels of complement component C5a, C3a, factor B and membrane attack complex (MAC) are seen in patients with active disease, and remission associates with their reduction [40]. C5a is a potent chemotactic factor for leukocytes and promotes activation and degranulation of neutrophils through its interaction with the C5a receptor, C5aR1, which is the target for the oral complement inhibitor avacopan (Fig. 3).

### Diagnosis

Prior to diagnosis of AAV, suspicion of a vasculitic illness is key and these disorders should be considered in patients with unexplained systemic or organ specific inflammation and/or dysfunction. Diagnostic delay remains an important contributor to poor outcomes especially for those with kidney involvement. The diagnosis of AAV involves a comprehensive approach that includes clinical manifestations, serologic testing [immunoassays for PR3‑ANCAs and MPO-ANCAs, eosinophils, exclusion of anti–glomerular basement membrane disease and systemic lupus erythematosus (SLE)] and when feasible tissue biopsy. Prompt treatment is often initiated in high-suspicion cases, while biopsy may still be performed later to confirm the diagnosis, exclude AAV mimics and guide further management, particularly from the kidney [41]. The International Chapel Hill Consensus Conference on the Nomenclature of Systemic Vasculitides and the subsequent ACR and EULAR have established classification criteria for AAV to aid in research and clinical studies, but are not designed to be diagnostic criteria [42–44].

### Outcome prediction

Disease-state definitions differentiate active disease from remission [22] and delays in achieving remission associate with higher mortality and ESKD risk [3]. Thus, monitoring for disease activity and response to treatment is key to the management AAV. Remission is defined by the absence of features of active disease while the patient is on a low prednisolone dose (usually ≤10 mg/day) following 3–6 months of immunosuppressive induction treatment. Renal remission is typically assessed by monitoring GFR, and urine abnormalities, particularly presence of haematuria. Persistent haematuria contributes to a higher risk of relapse while persistence of proteinuria (protein/creatinine ratio ≥0.05 g/mmol at 6 months) associates with worse long-term GFR [45, 46]. It is uncertain to what extent these urinary findings represent ongoing disease activity or persistent damage from the initial inflammation in the absence of repeat biopsy studies. New urine biomarkers such as soluble CD163 and MCP-1 have been investigated to gain insights into renal remission and treatment response [47]. An ongoing project by the ACR and EULAR is developing consensus composite response criteria for clinical trials [48].

Three clinicopathological risk scores, incorporating baseline parameters (clinical and pathological), have been developed to identify patients with renal involvement at risk of developing ESKD [49]. The Berden classification includes glomerular lesions (acute/chronic) and defines four classes; focal, mixed, crescentic and sclerotic, and the Mayo Clinic Chronicity score (MCCS) combines only the chronic features from all the renal compartments (glomeruli, interstitial, vessels) and presents four groups of severity. The ANCA Renal Risk Score (ARRS) incorporates both baseline kidney function, and histopathological characteristics (normal glomeruli and tubular atrophy/interstitial fibrosis), to predict the risk of ESKD categorized into three severity groups (recently updated to four groups) [50]. Although even in cases with the most chronic lesions or the highest risk of ESKD, there is still a chance of renal recovery, these scores cannot determine management decisions. However, further refinement has the potential for more precise medical interventions, for example histology findings to predict how patients will respond to specific drugs and/or PLEX.

Testing PR3/MPO-ANCA and/or CD19+ B cells for relapse prediction or guiding maintenance treatment duration can be helpful, but should not replace clinical assessment. There have been attempts to use these biomarkers to direct rituximab dosing which may allow a low total cumulative rituximab dose; however, frequency of monitoring, and sensitivity and specificity of assays limits ability to reliably prevent relapse [51]. Evidence suggests that ANCA levels and B-cell return maybe most helpful to predict relapse after rituximab withdrawal [52].

### Management

Rituximab is an anti-CD20 B-cell depleting monoclonal antibody that has proved effective in AAV as both induction and maintenance treatment; however, a small proportion of patients fail to achieve remission, especially in granulomatous disease, with the possibility of early relapses and rituximab resistance associated with an anti-globulin response. Obinutuzumab, a Type II anti-CD20 monoclonal antibody, has superior direct cell death and antibody-dependent cell-mediated cytotoxicity compared with rituximab and was efficacious in a phase II lupus nephritis trial, where more complete and sustained B-cell depletion occurred than has been observed in rituximab-treated SLE cohorts. The potential of obinutuzumab in AAV is being explored in Obinutuzumab compared with rituximab for treating ANCA-associated vasculitis (ObiVas) Trial (ISRCTN13069630). Failure to demonstrate a reduction of relapse risk with belimumab a BLyS inhibitor in the Efficacy and Safety of Belimumab and Azathioprine for Maintenance of Remission in Antineutrophil Cytoplasmic Antibody-Associated Vasculitis (BREVAS) Trial was linked to issues of trial design and a low event rate in the placebo group [53], but the potential for a synergistic B-cell depleting effect when belimumab is combined with rituximab from remission induction is being studied in the Randomised study of rituximab and belimumab sequential therapy in PR3 ANCA-associated vasculitis (COMBIVAS) [54].

Targeting T cells or T-cell help is also a rational approach in the treatment of AAV due to the crucial role of T cells the pathogenesis of the disease. Abatacept is comprised of the ligand-binding domain of CTLA4 plus human immunoglobulin and carries the potential to modulate the costimulatory signal required for T-cell activation. The promising results from an open-label trial of abatacept in non-severe GPA in terms of high rate of remission and its steroid-sparing effect has paved the way for the ongoing Abatacept (CTLA4-Ig) for the Treatment of Relapsing, Non Severe, Granulomatosis with Polyangiitis (Wegener's) (ABROGATE) Trial (NCT02108860).

The pivotal role of complement component C5a has addressed new target of treatment with avacopan and a newer anti-C5a monoclonal antibody (vilobelimab) which has brought promising results from phase 2 trial (abstract) [55]. Ongoing clinical trials with anti-IL5 agents [benralizumab (Efficacy and Safety of Benralizumab in EGPA Compared to Mepolizumab (MANDARA) Trial NCT04157348) and depemokimab (Efficacy and Safety of Depemokimab Compared With Mepolizumab in Adults With Relapsing or Refractory EGPA (OCEAN) Trial; NCT05263934)] will assess their effectiveness compared with mepolizumab in EGPA. Benralizumab and reslizumab have shown initial efficacy in small-open label pilot EGPA studies [56] (Fig. 3).

## SUMMARY

The updated EULAR recommendations for the management of AAV indicate significant progress in the field of AAV treatment. The direction towards a more effective treatment strategy with faster and higher rates of remission is of great importance in AAV management. Achieving remission promptly is vital for preventing disease progression, reducing organ damage and improving kidney outcomes. Advancements in understanding the underlying pathophysiology have led to the development of targeted therapies, such as B-cell depletion, complement inhibition, T-cell inhibition and anti-IL-5 inhibition, offering new opportunities for tailored treatments and improved disease management. The goal of eliminating relapses is also crucial in providing lasting disease control and preventing recurrent disease flares. The ultimate goal is to provide patients with AAV the best chance for sustained remission, kidney outcome, improved quality of life and reduced risk of relapses, while minimizing treatment-related side effects.

## Data Availability

No new data were generated or analysed in support of this research.
